# Myeloid Nrf2 Protects against Neonatal Oxidant-Stress-Induced Lung Inflammation and Alveolar Simplification in Mice

**DOI:** 10.3390/antiox13060698

**Published:** 2024-06-07

**Authors:** Chandra Mohan Tamatam, Lalith Kumar Venkareddy, Aparna Ankireddy, Narsa Machireddy, Sekhart P. Reddy

**Affiliations:** 1 Department of Pediatrics, University of Illinois, Chicago, IL 60612, USA; lalithvenkareddy@sduaher.ac.in (L.K.V.); aparna1@uic.edu (A.A.); nmreddy@northwestern.edu (N.M.); 2 Department of Cell Biology and Molecular Genetics, Sri Devaraj Urs Academy of Higher Education and Research, Tamaka, Kolar 563103, Karnataka, India; 3 Department of Pathology, The University of Illinois, Chicago, IL 60612, USA

**Keywords:** antioxidants, neonatal lung injury, bronchopulmonary dysplasia, macrophages

## Abstract

Bronchopulmonary dysplasia (BPD) is a chronic condition affecting preterm infants, characterized by lung alveolar simplification/hypoalveolarization and vascular remodeling. The nuclear factor erythroid 2 like 2 (Nfe2l2, or Nrf2) plays a critical role in the cytoprotective response to neonatal hyperoxia, and its global deficiency exacerbates hypoalveolarization in mice. The abnormal recruitment and activation of myeloid cells are associated with the pathogenesis of BPD. Therefore, we employed a genetic approach to investigate the role of myeloid Nrf2 in regulating hyperoxia-induced hypoalveolarization. Pups, both wild-type (*Nrf2*^f/f^) and those with a myeloid Nrf2 deletion (abbreviated as *Nrf2*^∆/∆mye^), were exposed to hyperoxia for 72 h at postnatal day 1 (Pnd1), and then sacrificed at either Pnd4 or Pnd18 following a two-week recovery period. We analyzed the hypoalveolarization, inflammation, and gene expression related to cytoprotective and inflammatory responses in the lungs of these pups. The hypoalveolarization induced by hyperoxia was significantly greater in *Nrf2*^∆/∆mye^ pups compared to their *Nrf2*^f/f^ counterparts (35.88% vs. 21.01%, respectively) and was accompanied by increased levels of inflammatory cells and IL-1β activation in the lungs. Antioxidant gene expression in response to neonatal hyperoxia was lower in *Nrf2*^∆/∆mye^ pups compared to their *Nrf2*^f/f^ counterparts. Furthermore, *Nrf2*-deficient macrophages exposed to hyperoxia exhibited markedly decreased cytoprotective gene expression and increased IL-1β levels compared to Nrf2-sufficient cells. Our findings demonstrate the crucial role of myeloid Nrf2 in mitigating hyperoxia-induced lung hypoalveolarization and inflammatory responses in neonatal mice.

## 1. Introduction

Bronchopulmonary dysplasia (BPD) is a chronic lung disease affecting preterm infants, characterized by alveolar simplification and impaired vascular growth, leading to lifelong morbidity and significant mortality. Although recent clinical approaches have improved the survival rates of preterm infants, the incidence of BPD remains high, with persistent poor clinical outcomes including an increased susceptibility to injurious insults and cognitive impairment [1,2]. Both preclinical and clinical research have implicated heightened oxidative stress and inflammation in the pathogenesis of BPD. Still, the specific contributions of lung resident and non-resident immune cells are not fully understood.

The nuclear factor erythroid 2 like 2 (Nfe2l2, or Nrf2) mitigates cellular stress by upregulating gene expression via the antioxidant response element (ARE), protecting the lung and other organs from infectious and non-infectious injuries [3,4]. While not essential for development, Nrf2 is vital for the induction of antioxidant gene expression and for the gene expression involved in lung cell regeneration after tissue injury [5]. Our studies have shown that Nrf2-deficient mice exhibit persistent inflammation during their recovery from sub-lethal oxidant-induced lung injury. Additionally, the genetic disruption of Nrf2 exacerbates hyperoxic lung injuries in neonatal mice, leading to worsened alveolar simplification or hypoalveolarization [6,7]. Recently, we demonstrated that preconditioning immature lungs with the genetic activation of Nrf2 target gene expression prevents oxidant-induced alveolar simplification in vivo [8].

Myeloid cells are a significant cell type in the developing lung, and their abnormal recruitment or activation following toxicant and oxidant exposure can impair normal lung development and promote pathogenesis [9]. However, the mechanisms through which myeloid cells contribute to BPD pathogenesis, particularly in regulating neonatal oxidative stress, still need to be fully understood. We hypothesize that the Nrf2 in myeloid cells is crucial for mitigating the lung inflammation and hypoalveolarization induced by neonatal hyperoxia, potentially serving as a therapeutic target for BPD. Here, we investigated the specific role of myeloid Nrf2 in regulating oxidant-induced alveolar simplification in vivo. We demonstrate that this transcription factor in myeloid cells is crucial for dampening lung inflammation and alveolar simplification. Activating macrophage Nrf2 as a therapeutic target may help accelerate the repair of stunted alveolar and vascular growth in preterm infants.

## 2. Materials and Methods

### 2.1. Neonatal Hyperoxia Exposure

Details of the *Nrf2*^−/−^ mice have been described elsewhere [10]. Mice bearing “floxed” *Nrf2* alleles (*Nrf2*^f/f^) were crossed with mice bearing LysM-Cre to delete the *Nrf2* in their myeloid cells (hereafter referred to as *Nrf2*^∆/∆mye^). Newborn (Pnd1) pups from WT (*Nrf2*^+/+^), *Nrf2*^−/−^, *Nrf2*^f/f^, and *Nrf2*^∆/∆mye^ mice were exposed to hyperoxia (95% oxygen) for 72 h, with rotating dams every 24 h. Pups exposed to room air (RA) were used as controls. The Nrf2-gene-specific primers forward 5′-TCT TAG GCA CCA TTT GGG AGA G-3′ and reverse 5′-TAC AGC AGG CAT ACC ATT GTG G-3’ were used to identify “floxed” (750 bp) and wild-type (500 bp) alleles.

### 2.2. Lung Morphometry

Alveolar simplification was assessed as previously detailed [8]. Briefly, mice were sacrificed, and their left lung was inflated with agarose and 1.5% paraformaldehyde, excised, washed, and kept in 70% ethanol. The lung was sectioned (5 μm) and stained with hematoxylin and eosin (H&E). Slides were coded, and lung images were captured with a Nikon Digital Camera Nikon, Tokyo, Japan, or with an Aperio AT2^®^ scanner (Leica Biosystems, Deer Park, IL, USA) at ×10. The mean chord length (MCL) was calculated by analyzing digital JPEG images using lung STEPanizer1 software (www.stepanizer.com), excluding arteries, veins, and bronchioles.

### 2.3. Lung Inflammation

The bronchoalveolar lavage (BAL) fluid was obtained by lavaging the right lung twice with 0.5 mL PBS with 1 mM EDTA; the BAL fluid was centrifuged, and the total number of lung cells were counted. Cells were cytospinned, stained with a Diff-Quick Stain Set (Thermo Scientific, Waltham, MA, USA), and enumerated for differential counting.

### 2.4. Real-Time PCR Analysis

RNA from the lung tissue was isolated, cDNA was prepared using a qScript cDNA Synthesis Kit (Cat# 4385612, Thermo Scientific, Waltham, MA, USA), and a Fast SYBR green quantitative real-time PCR (qRT-PCR) assay (Cat# 4385612, Applied Biosystems, Waltham, MA, USA) was performed using target-gene-specific primers (Supplemental Table S1). β-actin was used as a control to normalize and calculate the relative gene expression levels.

### 2.5. Lung Macrophage Isolation and BMDM Culture

Lung macrophages were obtained from the BAL tissue fluid and plated on culture dishes for 30 min. The supernatant was removed, and the cells were lysed in a DNA extraction buffer for the genotyping analysis. Bone marrow-derived macrophages (BMDMs) were prepared from WT mice (WT-BMDMs) and *Nrf2*^−/−^ mice (NKO-BMDMs) using standard approaches. Briefly, bone marrow cells were lysed in ACK lysing buffer (Lonza, Rockville, MD, USA) to remove red blood cells and centrifuged. The cell pellet was resuspended and cultured in RPMI-1640 with 10% FBS and conditioned L929 medium. The cell culture medium was replenished on alternate days for 5–6 days. The cells were lifted and plated in equal numbers (~1 million cells) overnight before use in the experiments.

### 2.6. Statistical Analysis

For multiple comparisons, a two-way analysis of variance with Tukey’s post hoc test was performed using GraphPad Prism. Student’s *t*-test was used for comparisons between the two groups. The symbols *, **, ***, and ****, and ^$^, ^$$^, ^$$$^, and ^$$$$^ shown in the figures represent *p* values < 0.05, 0.01, 0.001, and 0.0001, respectively. *, means compared to the RA controls of the respective genotypes; and ^$^, means compared to *Nrf2*^+/+^ (WT) counterparts and *Nrf2*^f/f^ counterparts.

## 3. Results

### 3.1. Global Nrf2 Deficiency Increases Susceptibility to Neonatal Hyperoxia-Induced Alveolar Simplification

**Figure 1:**
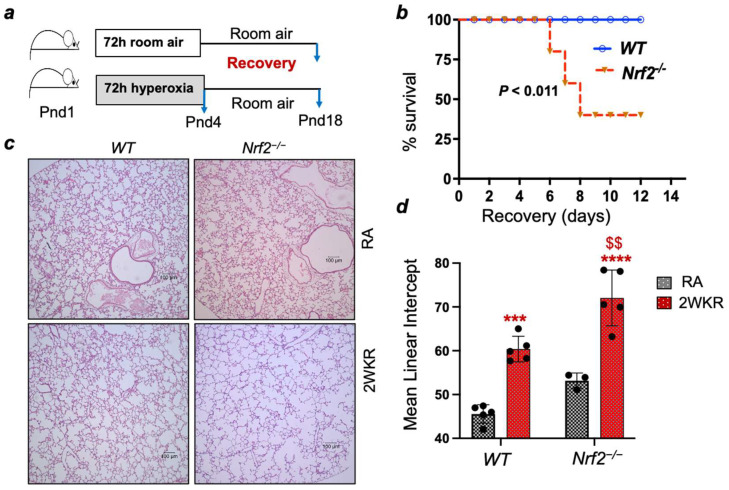
(**a**) Wild-type (Nrf2^+/+^) and Nrf2^–/–^ pups at Pnd1 were exposed to hyperoxia for 72 h and recovered at room air for 14 days as outlined. (**b**) Survival rates of neonatal hyperoxia-exposed Nrf2^+/+^ and Nrf2^–/–^ pups in recovery. (**c**) Room air (RA) and 72 h hyperoxia-exposed and recovered Nrf2^+/+^ and Nrf2^–/–^ pups at Pnd18 (2WKR) were sacrificed, and the left lung was fixed, sectioned and stained with H&E. A representative image of the lung sections is shown (20×). (**d**) Mean chord length (MCL) of the alveolar region of H&E images (10×) was analyzed by morphometry, as detailed in the methods. ***, ****, compared to respective RA controls; ^$$^, compared to Nrf2^+/+^ counterparts. Scale bars: 100 µm.

Newborn (Pnd1) pups’ lungs resemble the saccular developmental stage of premature babies. The exposure of Pnd1 pups to 95–100% oxygen for 72 h, followed by their recovery in room air for one to two weeks, or their chronic exposure to <85% oxygen for 10-14 days, leads to alveolar simplification and vascular remodeling [11]. Nrf2^−/−^ mice are susceptible to infectious and non-infectious acute lung injury as adults, but their susceptibility to oxidant injury differs between pups and adult mice [5]. Therefore, to determine whether a Nrf2 deficiency affects lung development after pro-oxidant exposure, Nrf2^+/+^ and Nrf2^−/−^ pups at Pnd1 were exposed to 95% oxygen for 72 h and then left to recover for two weeks (referred to as 2WKR) (Figure 1a). We chose this subacute hyperoxia exposure model of BPD as the continuous chronic exposure of Nrf2^−/−^ pups to hyperoxia results in mortality. Moreover, this model will help define the mechanisms of neonatal lung injury’s resolution and repair. As shown in Figure 1b, hyperoxic stress caused significant mortality in the Nrf2^−/−^ pups recovering in room air, while all Nrf2^+/+^ pups survived. To assess the effects of Nrf2 deficiency on lung development, the Nrf2^−/−^ pups that survived hyperoxic stress during recovery were sacrificed, and their left lungs were fixed, sectioned, and used for morphometric analysis. Histopathology revealed increased alveolar simplification in the Nrf2^−/−^ pups compared to the Nrf2^+/+^ mice at the 2-week recovery period (Figure 1c). We then evaluated the degree of their mean chord length (MCL) as a phenotypic marker of alveolar simplification/enlargement between room air and hyperoxia-exposed Nrf2^+/+^ and Nrf2^−/−^ pups (Figure 1d). Consistent with the histopathology, the MCL quantification revealed an increased level of alveolar simplification in the Nrf2^−/−^ pups exposed to hyperoxia compared to their Nrf2^+/+^ counterparts (Figure 1d). These results suggest that Nrf2 is necessary to mitigate the adverse effects of neonatal hyperoxia during recovery.

### 3.2. Myeloid Nrf2 Deficiency Worsens Neonatal Hyperoxia-Induced Alveolar Simplification

**Figure 2:**
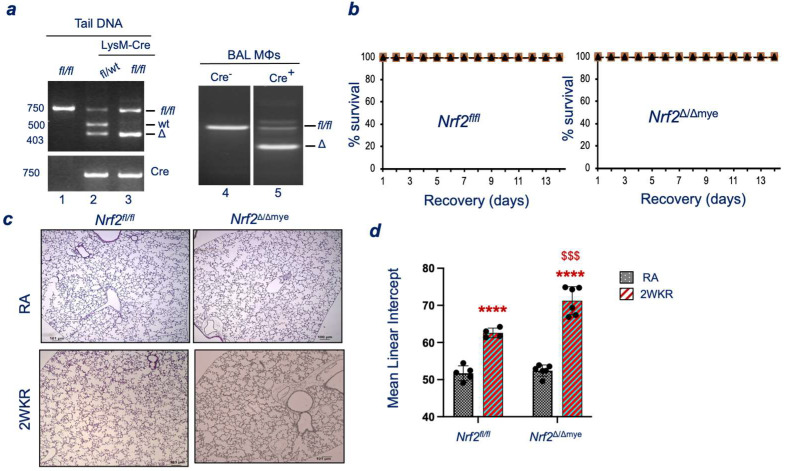
(**a**) Mice with Nrf2 deletion in myeloid cells (Nrf2^Δ/Δmye^) were generated by crossing Nrf2-floxed mice with LysM2-Cre mice and genotyped. Nrf2 deletion in Nrf2^Δ/Δmye^ mice was characterized in tail DNA and BAL lung macrophages (MΦs) by PCR genotyping. (**b**) Newborn (Pnd1) pups from Nrf2^f/f^ (left panel) and Nrf2^Δ/Δmye^ mice (right panel) were exposed to 72 h hyperoxia and then recovered at room air for two weeks (2WKR), as in Figure 1a. Their survival rates were monitored for up to 14 days. (**c**) Pups exposed to room air or 72 h hyperoxia and sacrificed at Pnd18, and the left lung was fixed, sectioned, and stained with H&E. A representative image of the lung sections of both genotypes is shown (20×). (**d**) H&E images (10×) were analyzed to determine the mean chord length (MCL). ****, compared to respective genotype RA controls; ^$$$^, compared to Nrf2^f/f^ counterparts. Scale bars: 100–101 µm.

Myeloid cells constitute a major cell type in the developing murine lung, and their abnormal recruitment or activation severely affects normal lung development and oxidant-induced pathogenesis [9]. To determine whether the Nrf2-mediated signaling in myeloid cells regulates oxidant-induced alveolar simplification, Nrf2-floxed mice (Nrf2^f/f^) were bred with mice bearing Cre recombinase under the control of the LysM promoter (see schema). Due to this breeding, Nrf2^f/f^ mice bearing LysM-Cre lack Nrf2 primarily in their myeloid cells (hereafter referred to as Nrf2^∆/∆mye^). As shown in Figure 2a, we have verified the deletion of Nrf2 in the lungs of Nrf2^∆/∆mye^ mice. Newborn (Pnd1) pups from Nrf2^f/f^ (left panel) and Nrf2^∆/∆mye^ mice (right panel) were exposed to room air or 95% oxygen for 72 h and recovered at room air. The pups’ mortality was monitored Figure 2b). In contrast to the Nrf2^−/−^ pups (Figure 1b, see above), all Nrf2^∆/∆mye^ pups survived recovery following neonatal hyperoxia (Figure 2b, right panel). Histopathology (Figure 2c) and a quantitative morphometry analysis (Figure 2d) revealed alveolar simplification in Nrf2^f/f^ pups. However, this phenotype was more predominant in Nrf2^∆/∆mye^ pups than in their Nrf2^f/f^ counterparts (21.01% vs. 35.88%, Nrf2^f/f^ vs. Nrf2^∆/∆mye^). Notably, the extent to which neonatal hyperoxia increased the MCL in Nrf2^∆/∆mye^ pups (Figure 2d) was comparable to that seen in their Nrf2^−/−^ counterparts (Figure 1d). These results demonstrate that Nrf2 signaling in myeloid cells is essential for dampening hyperoxia-induced alveolar simplification, but its deficiency is not detrimental.

### 3.3. Myeloid Nrf2 Deficiency Increases Lung Inflammation following Neonatal Hyperoxia

**Figure 3:**
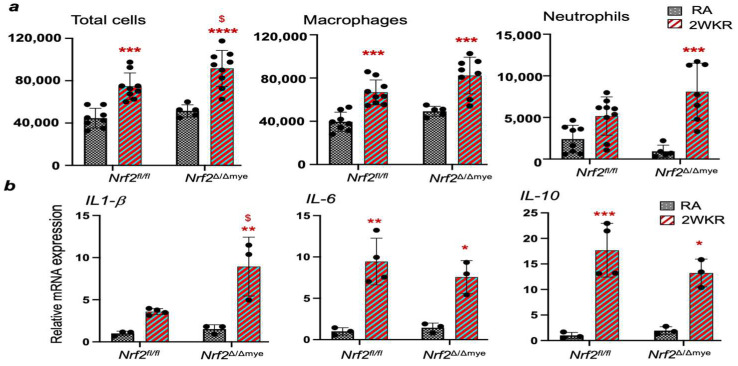
(**a**) Room air or neonatal hyperoxia-exposed Nrf2^f/f^ and Nrf2^Δ/Δmye^ pups were sacrificed at Pnd18, the right lung was lavaged, and inflammatory cells were enumerated. (**b**) RNA was isolated from the lungs of Nrf2^f/f^ and Nrf2^Δ/Δmye^ pups as detailed in panel A, and cytokine gene expression was analyzed by qPCR. *, **, ***, ****, compared to respective RA controls; ^$^, compared to Nrf2^f/f^ counterparts.

We next examined whether the worsened alveolar simplification in Nrf2^∆/∆mye^ pups is accompanied by increased lung inflammation. Mice were sacrificed at Pnd18, and their right lung was used for the bronchoalveolar lavage (BAL) fluid collection and inflammatory cell analysis, while the left lung was formalin-fixed for a morphometry analysis, as detailed above. As illustrated in Figure 3a, the BAL cell analysis revealed increased levels of inflammatory cells in the lungs of hyperoxia-exposed mice compared to their vehicle-treated counterparts. In the Nrf2^∆/∆mye^ pups, the total cells that were macrophages and neutrophils were higher than in their WT counterparts, suggesting an increased inflammatory response in these mice. We also determined cytokine gene expression levels to ascertain whether Nrf2 deficiency affects lung inflammation (Figure 3b). Indeed, we found increased IL-1β, IL-6, and IL-10 expression in the lungs of the Nrf2^f/f^ and Nrf2^∆/∆mye^ pups compared to their room air counterparts. However, the IL-1β expression was significantly greater in the Nrf2^∆/∆mye^ pups compared to their Nrf2^f/f^ counterparts.

### 3.4. Myeloid Nrf2 Deficiency Results in Decreased Cytoprotective and Heightened Cytokine Gene Expression in Response to Neonatal Hyperoxia

**Figure 4:**
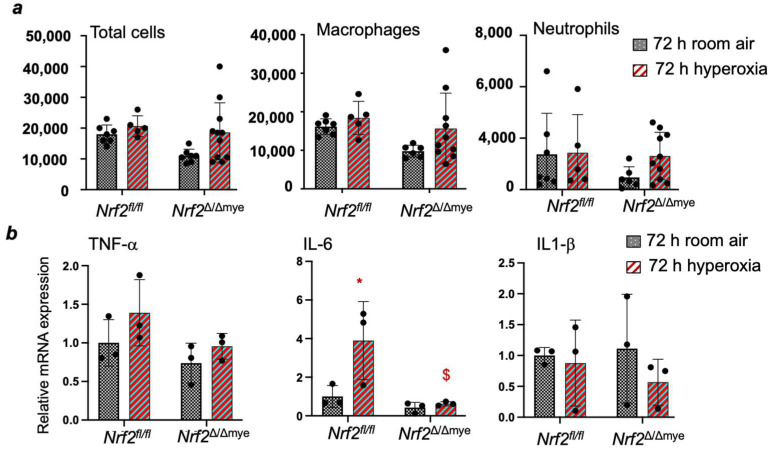
(**a**) *Nrf2*^Δ/Δmye^ and *Nrf2*^f/f^ pups were exposed to hyperoxia for 72 h and immediately sacrificed; the right lung was lavaged, and inflammatory cells were assessed. (**b**) RNA was isolated from the lungs of *Nrf2*^Δ/Δmye^ and *Nrf2*^f/f^ pups exposed to 72 h hyperoxia, and target gene expression was analyzed by qPCR. *, compared to respective RA controls, ^$^, compared to *Nrf2*^f/f^ counterparts.

**Figure 5:**
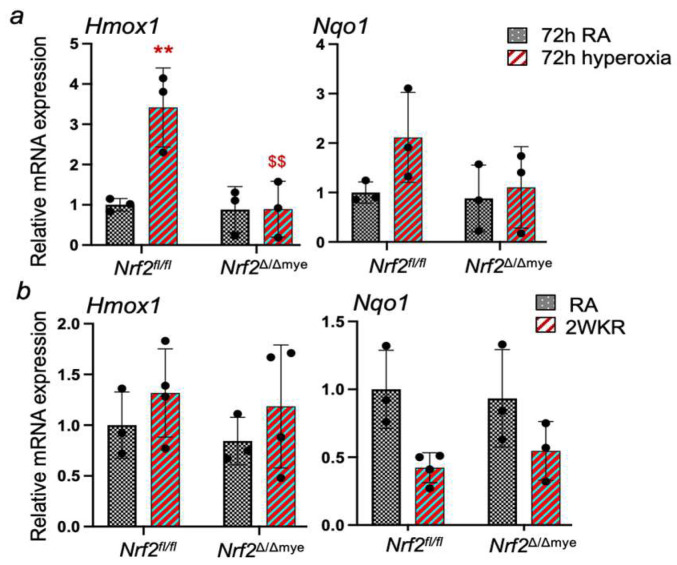
RNA was isolated from the lungs of *Nrf2*^Δ/Δmye^ and *Nrf2*^f/f^ pups exposed to neonatal hyperoxia and recovered at room air for 14 days (**a**) from the lungs of *Nrf2*^Δ/Δmye^ and *Nrf2*^f/f^ pups exposed to neonatal hyperoxia for 72 h and (**b**) Nrf2 putative target gene expression was analyzed by qPCR. **, compared to respective RA controls; ^$$^, compared to *Nrf2*^f/f^ counterparts.

We have analyzed whether myeloid Nrf2 regulates inflammatory cytokine and cytoprotective antioxidant gene expression in neonatal lungs in response to hyperoxia. As shown in Figure 4a, we did not find significant levels of hyperoxia-induced lung inflammation in the BAL fluids of Nrf2^f/f^ and Nrf2^∆/∆mye^ pups. There were no significant differences in the levels of inflammatory macrophages or neutrophils between the two genotypes. In agreement with this result, the qPCR analysis showed a lack of a robust proinflammatory IL-1β and TNFα induction in the lungs of both genotypes (Figure 4b). However, IL-6 expression was induced by hyperoxia in the WT pups but not in the myeloid Nrf2-deficient pups. Next, we examined the Nrf2 putative antioxidant gene (Hmox1 and Nqo1) expression levels in these pups. Increased levels of Hmox1 and Nqo1 were observed in the lungs of hyperoxia-exposed Nrf2^f/f^ pups (Figure 5a). However, their expression levels were not heightened in Nrf2^∆/∆mye^ pups.

**Figure 6:**
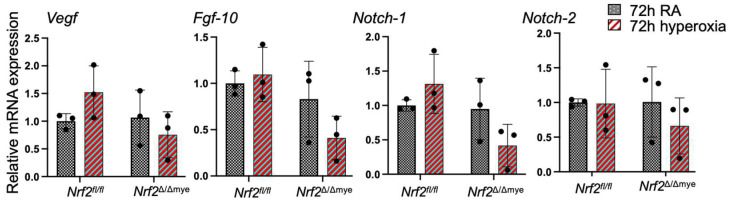
RNA isolated from the lungs of *Nrf2*^Δ/Δmye^ pups and *Nrf2*^f/f^ pups exposed to room air (RA) or 72h of hyperoxia at Pnd1 and sacrificed at Pnd4 was analyzed for Vegf, Fgf10, Notch1, and Notch2.

We also analyzed Hmox1 and Nqo1 expression in the lungs of mice who had recovered from hyperoxia exposure (Figure 5b). The Hmox1 and Nqo1 expression levels were not significantly altered in both genotypes compared to their room air counterparts, though Nqo1 showed a modestly reduced expression in Nrf2-deficient pups. We also analyzed whether myeloid Nrf2-deficiency affects growth factor gene expression. Vegf, Fgf-10, Notch1, and Notch2 gene expression was not altered by hyperoxia, and there were no significant differences in their expression levels between the Nrf2^f/f^ and Nrf2^∆/∆mye^ pups (Figure 6).

### 3.5. Nrf2 Regulates the Cytoprotective and Cytokine Gene Expression in Macrophages in Response to Hyperoxia

**Figure 7:**
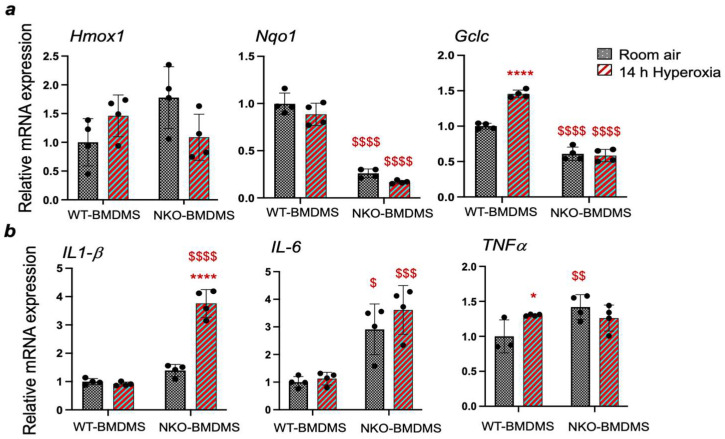
Freshly cultured BMDMs isolated from *Nrf2*^+/+^ (WT) and *Nrf2*^–/–^ (NKO) mice were exposed to room air (RA) or hyperoxia for 14 h, and RNA was isolated. Both cytoprotective (**a**) and inflammatory cytokine (**b**) gene expression were determined by qPCR using gene-specific primers as indicated. *, ****, compared to respective genotype RA controls; ^$^, ^$$^, ^$$$^, ^$$$$^, compared to WT-BMDMs.

To determine whether Nrf2 regulates the antioxidant and inflammatory cytokine gene expression in macrophages in response to hyperoxia, wild-type/Nrf2^+/+^ BMDMs (WT-BMDMs) and Nrf2^−/−^ BMDMs (NKO-BMDMs) were exposed to hyperoxia and their gene expression was evaluated by qPCR (Figure 7). As anticipated, the Nrf2 putative Nqo1 and Gclc expression in Nrf2^−/−^ BMDMs in their basal state was significantly lower than in Nrf2^+/+^ BMDMs. Hyperoxia stimulated Gclc expression in wild-type cells but not in Nrf2^−/−^ BMDMs. At the same time, hyperoxia did not affect Hmox1 and Nqo1 expression levels (Figure 7a). We noted significantly increased levels of IL-6 and TNFα expression in Nrf2^−/−^ BMDMs in their basal state (Figure 7b), but hyperoxia did not affect their expression in both genotypes. However, we found markedly increased levels of IL-1β in hyperoxia-exposed Nrf2^−/−^ BMDMs compared to their Nrf2^+/+^ counterparts (Figure 7b).

## 4. Discussion

The present study demonstrates that myeloid Nrf2 signaling is crucial to restricting neonatal hyperoxia-induced hypoalveolarization, a significant human BPD phenotype associated with prematurity. This transcription factor deficiency in myeloid cells results in the decreased induction of cytoprotective genes due to neonatal hyperoxia and subsequently causes heightened lung inflammation with worsened hypoalveolarization during the recovery from injury. The degree of worsened hyperoxia-induced hypoalveolarization in *Nrf2*^∆/∆mye^ pups is comparable to that in *Nrf2*-null pups, demonstrating the predominant role of myeloid Nrf2 signaling in diminishing the severity of oxidant stress-induced BPD pathogenesis. However, unlike *Nrf2*-null mice (Figure 1b), which exhibit mortality [6,7], the deficiency of this transcription factor in their myeloid cells does not result in mortality during recovery (Figure 2b), suggesting that Nrf2 signaling in other cell types mitigates the detrimental effects of neonatal hyperoxia.

The myeloid cell population, consisting of macrophages and neutrophils, plays a role in the development of the lung and in regulating lung repair and pathogenesis after injury [1,12,13,14]. Macrophages have divergent roles in modulating neonatal lung inflammation and pathogenesis. For example, CD11b^+^ monocytes recruited to the lung offer protection from hyperoxia-induced neonatal lung injury [15], whereas blocking CCR2+ monocytes protects from LPS-induced hypoalveolarization [16]. Our findings indicate that myeloid Nrf2 acts as an upstream endogenous mediator, attenuating the heightened lung inflammation and alveolar simplification induced by neonatal hyperoxia. The oxidant-induced activation of macrophages in the developing lung results in an altered lung structure with alveolar simplification [17]. For example, NF-κB and IL-1β/inflammasome activation by toxicant or inflammatory stimuli triggers abnormal fetal lung development and BPD pathogenesis, and targeting proinflammatory mediators (e.g., IL-1β, IL-6) using genetic- and antibody-based approaches blocks neonatal lung pathogenesis [18,19,20,21,22].

Interestingly, *Nrf2*^∆/∆mye^ pups in recovery from hyperoxic injury showed increased alveolar simplification accompanied by lung neutrophilic inflammation and IL-1β activation. We observed a decreased expression of antioxidant genes (Nqo1, Hmox1) in the lungs of hyperoxia-exposed *Nrf2*^∆/∆mye^ pups compared to their *Nrf2*^f/f^ counterparts; however, their expression levels did not change in recovered pups. While our findings suggest the involvement of myeloid Nrf2 in restricting oxidant-induced alveolar simplification, whether this transcription factor regulates lung macrophage plasticity or limits inflammatory cytokine gene expression directly or indirectly by modulating antioxidant gene expression in the neonatal lungs warrants further investigation.

Inflammation contributes to neonatal hyperoxia-induced lung pathogenesis, with IL-1β activation playing a significant role in this process. Our studies demonstrate that increased levels of IL-1β expression, consistent with previous reports, are associated with worsening lung inflammation and alveolar simplification in *Nrf2*^∆/∆mye^ pups. Elevated levels of IL-6 and IL-1β have been observed in BPD patients [23,24]. Hyperoxia activates IL-6 in macrophages, and IL-6 impairs alveolar type II cell homeostasis and disrupts elastic fiber formation, thereby inhibiting lung growth in vivo [19]. However, we found that the IL-6 expression in the lungs of *Nrf2*^∆/∆mye^ pups recovered from neonatal hyperoxia was comparable to that of their WT counterparts (Figure 3). Interestingly, IL-6 expression was markedly lower in *Nrf2*^∆/∆mye^ pups than in WT pups exposed to acute hyperoxia (Figure 4). Macrophages lacking Nrf2 showed increased levels of IL-6 expression in their basal state. Still, hyperoxia did not affect its expression in both genotypes (Figure 7). While further studies are warranted to address the role of temporal and variable IL-6 expression, our data suggest that this cytokine may not play a role in worsened oxidant-induced alveolar simplification in the setting of myeloid Nrf2 deficiency.

Despite significant Nrf2 target gene induction, wild-type (*Nrf2*^+/+^) pups develop hypoalveolarization after hyperoxic injury, suggesting inadequate Nrf2 target gene induction to alleviate cellular stress and promote neonatal lung repair. Indeed, our research demonstrates that increasing endogenous Nrf2 activity by the global genetic disruption of Keap1, the primary endogenous inhibitor of Nrf2, mitigates neonatal hyperoxia-induced alveolar simplification [8]. The pharmacological activation of Nrf2 reduced oxidative stress levels, and NLRP3/IL-1β inflammasome activation was linked to hypoalveolarization in mice [25]. Therefore, further studies are necessary to ascertain whether targeting the Keap1/Nrf2 in myeloid cells would aid in alleviating the hypoalveolarization associated with prematurity.

## 5. Conclusions

Our studies demonstrate that myeloid Nrf2 is crucial in regulating the alveolar simplification in neonatal mice induced by oxidant stress. Nrf2 activity in myeloid cells is necessary to mitigate oxidant-stress-induced hypoalveolarization by upregulating antioxidant gene induction during neonatal hyperoxia and decreasing the inflammatory response during recovery. Our current findings lay the framework for increasing myeloid Nrf2 activity as a therapeutic target for mitigating the alveolar simplification associated with prematurity.

## Data Availability

The data are available upon a reasonable request.

## Supplementary Materials

The following supporting information can be downloaded at: https://www.mdpi.com/article/10.3390/antiox13060698/s1, Table S1: Primers used for qRT-PCR analysis.
